# Single Cell Analysis of Inertial Migration by Circulating Tumor Cells and Clusters

**DOI:** 10.3390/mi14040787

**Published:** 2023-03-31

**Authors:** Jian Zhou, Alexandra Vorobyeva, Qiyue Luan, Ian Papautsky

**Affiliations:** 1 Department of Biomedical Engineering, University of Illinois Chicago, Chicago, IL 60607, USA; 2 UI Cancer Center, University of Illinois Chicago, Chicago, IL 60607, USA

**Keywords:** circulating tumor cells, CTC clusters, CTC doublets and triplets, inertial microfluidics, inertial migration, single-cell analysis, cell focusing and separation

## Abstract

Single-cell analysis provides a wealth of information regarding the molecular landscape of the tumor cells responding to extracellular stimulations, which has greatly advanced the research in cancer biology. In this work, we adapt such a concept for the analysis of inertial migration of cells and clusters, which is promising for cancer liquid biopsy, by isolation and detection of circulating tumor cells (CTCs) and CTC clusters. Using high-speed camera tracking live individual tumor cells and cell clusters, the behavior of inertial migration was profiled in unprecedented detail. We found that inertial migration is heterogeneous spatially, depending on the initial cross-sectional location. The lateral migration velocity peaks at about 25% of the channel width away from the sidewalls for both single cells and clusters. More importantly, while the doublets of the cell clusters migrate significantly faster than single cells (~two times faster), cell triplets unexpectedly have similar migration velocities to doublets, which seemingly disagrees with the size-dependent nature of inertial migration. Further analysis indicates that the cluster shape or format (for example, triplets can be in string format or triangle format) plays a significant role in the migration of more complex cell clusters. We found that the migration velocity of a string triplet is statistically comparable to that of a single cell while the triangle triplets can migrate slightly faster than doublets, suggesting that size-based sorting of cells and clusters can be challenging depending on the cluster format. Undoubtedly, these new findings need to be considered in the translation of inertial microfluidic technology for CTC cluster detection.

## 1. Introduction

Circulating tumor cell (CTC) clusters have been attracting attention due to their clinical significance and critical role in cancer metastasis [1,2,3,4]. CTC clusters are aggregates of two or more cells shed from primary tumors into the blood circulation [1,3,5]. The presence of CTC clusters has been associated with worse clinical outcomes as compared with that of single CTCs [6,7]. CTC clusters are more aggressive with 20–100 times higher metastatic potential [1,6,8]; however, very little is known about the mechanistic formation of CTC clusters [3]. One of the main reasons is the lack of access to these metastatic precursors [9]. Single CTCs are already rare events in circulation, as there can be only one CTC per billions of blood cells [10,11]; however, CTC clusters can be 10 times less frequent than single CTCs [10,12]. This extreme scarcity of CTC clusters has been a critical challenge in developing effective isolation technologies [10,13].

Inertial microfluidics is one technology that has been successful in the isolation of CTCs and clusters [10,14,15,16,17]. Cells inside an inertial flow are subjected to inertial forces that lead to the lateral migration of cells [18,19,20]. This lateral migration is highly size-dependent and thus the separation can be achieved without labeling due to the size difference between CTCs and blood cells [15,20]. This technology is very promising for the detection of rare cells as no additional labeling is required and the separation process is continuous with high throughput [14]. We and others have demonstrated the isolation of CTCs and clusters from the samples of non-small cell lung carcinoma (NSCLC) [21,22], head and neck cancer (HNC) [23,24], hepatocellular carcinoma (HCC) [17,25], and breast cancer (BCa) [26,27,28,29] patients using inertial microfluidic devices. Work to date has focused on the separation of single CTCs from blood cells. The separation of CTC clusters from single cells has rarely been investigated. The common assumption based on the migration behavior of rigid spherical particles is that the separation should be readily achievable as the clusters are larger than single cells. However, the inertial migration behavior of cell clusters remains unclear.

In this work, we investigate the inertial migration behavior of individual cell clusters using high-speed imaging. Clusters of A549 cells of lung cancer origin were formed in a low-adherent well plate. Considering the real-world application of sorting live cells, we used live cell clusters suspended in PBS without fixation. Our early work [20] indicates that inertial migration is fastest when the Reynolds number (Re) is around 50. To maximize the lateral displacement in the limited field of view (~1 mm) of our microscope, cell clusters were run at Re = 48 in a straight microchannel with a rectangular cross-section of 160 µm × 50 µm, which is sufficient to avoid the potential clogging due to cell clusters. Since doublets and triplets are the most common formats of cell clusters [30,31], we specifically investigated the inertial migration behavior of these clusters as well as the single cells as a control group. Unexpectedly, we found that triplets do not migrate faster than doublets despite triplets being larger than doublets. We found the two formats of the triplets, namely string triplets and triangle triplets, were different in migration behavior. These unexpected findings are against the common understanding of the size-dependent migration of inertial microfluidics. We expect these findings are critical factors to be considered when developing inertial microfluidic devices for sorting tumor cell clusters.

## 2. Experimental Methods

### 2.1. Device Fabrication and Experimental Setup

Microchannels were fabricated in polydimethylsiloxane (PDMS) using a dry film master. The process for making the dry film master is detailed in our recent work [32]. Briefly, a 160 µm × 50 µm rectangular straight microchannel was patterned on a 3” silicon wafer using dry films (ADEX 50, DJ MicroLaminates Inc., Sudbury, MA, USA). The microchannel was then replicated in PDMS (Sylgard 184, Dow Corning^®^, Midland, MI, USA) slabs, which was bonded to 1” × 3” glass slides (Fisher Scientific, Waltham, MA, USA) to form sealed devices after oxygen surface plasma treatment (PE-50, Plasma Etch Inc., Carson City, NV, USA) for 20 s. Inlet and outlet holes were manually punched using a biopsy punch with an outer diameter of 1.5 mm (Ted Pella Inc., Redding, CA, USA). Cell sample solution was loaded in a syringe (Norm-Ject^®^, Air-Tite Co Inc., Berwyn, IL, USA), which was connected to 1/16” Tygon^®^ tubing (Cole-Palmar, Vernon Hills, IL, USA) using proper fittings (IDEX Health & Science LLC, Oak Harbor, WA, USA). The other end of the tubing was secured to the device inlet. A syringe pump (Legato 200, KD Scientific Inc., Holliston, MA, USA) was used to sustain stable flow rate of 300 µL/min (Re = 48), which maximizes the lateral displacement for high-speed imaging in 1 mm field of view using a 20× objective [33]. The microchannel was placed on the stage of an inverted microscope (IX83, Olympus America, Center Valley, PA, USA).

### 2.2. Cell Sample Preparation

Non-small-cell-lung cancer (NSCLC) cell line A549 (ATCC, Manassas, VA, USA) was cultured in RPMI 1640 medium (Fisher Scientific, Waltham, MA, USA) supplemented with 10% (*v*/*v*) FBS (GeminiBio Inc., West Sacramento, CA, USA), and 1% (*v*/*v*) 100× antibiotic–antimycotic solution (Invitrogen, Carlsbad, CA, USA) in an incubator at 37 °C and 5% CO_2_. Cell aggregates were formed in the low attachment plates, which were made by coating 12-well plates with anti-adherence solution (Stemcell Technology, Vancouver, Canada). Anti-adherence solution was spread across the entire well bottom and excess was removed. Plates treated with anti-adherence solution were placed in a biosafety hood overnight under UV exposure until they completely dried. Then, 1 mL of A549 cell suspension was added into each well at 500k cells/mL. After 2 days of culture, cell suspensions containing single cells, doublets, triplets, and other cell aggregates were re-suspended in phosphate-buffered solution (PBS) to reach final concentration of 2.2 × 10^4^ objects (cells or aggregates) per mL before they were run into the microchannel. Cell viability was 93 ± 8%, which was determined by double staining of calcein-AM and propidium Iodide (PI) (Fisher Scientific, Waltham, MA, USA).

### 2.3. Image Acquisition and Data Analysis

Images of single cells, doublets, and triplets were acquired using a high-speed camera (Mini AX200, Photron USA Inc., San Diego, CA, USA). The frame rate was 25,000 fps and exposure time was 1 µs to ensure no distortion of the moving cells. PFV software (version 3, Photron USA Inc., San Diego, CA, USA) was used along with high-speed camera in obtaining more than 8000 frames a time. The field of view was 1024 µm × 256 µm using 20× high NA objective (NA = 0.7). Raw images were converted into TIFF files before they were analyzed in ImageJ^®^. Image stacks of individual cells, doublets, and triplets were manually extracted from the raw data using “Make Substack” module in ImageJ^®^. Threshold of each stack was properly set before the analysis using “Analyze Particle” module in ImageJ^®^. Multiple parameters such as the coordinates of the center of the cell or aggregate, area, angle, aspect ratio (AR), and others were extracted for each frame. The coordinates of the cell or aggregate center were in sub-micron accuracy as the center was calculated based on the cell or aggregate outline. Based on the frame rate (FR) and the displacements in downstream (x-direction) and lateral (y-direction) directions, the downstream and lateral migration velocities of a single cell, doublet, or triplet were calculated. Note that the downstream velocities calculated are the average velocity between the first frame and last frame of the image stack and the lateral migration velocity (V_m_) is the average velocity between the first frame and the frame of concern, for example, V_m_ = (y*_i_* − y_1_)/((i − 1)/FR). The angle measured (angle between the primary axis of the object and the downstream direction in *x*-axis) was about the *z*-axis in this work since the images were in xy plane. The comparison of velocities of singlets, doublets, and triplets was implemented in Originlab (version 2021b, OriginLab Corporation, Wellesley Hills, MA, USA) using one-way ANOVA (analysis of variance) and Tukey’s statistical method. Significant difference was defined as * *p* < 0.05.

## 3. Results and Discussion

### 3.1. Inertial Migration of Single Cells

**Figure 1:**
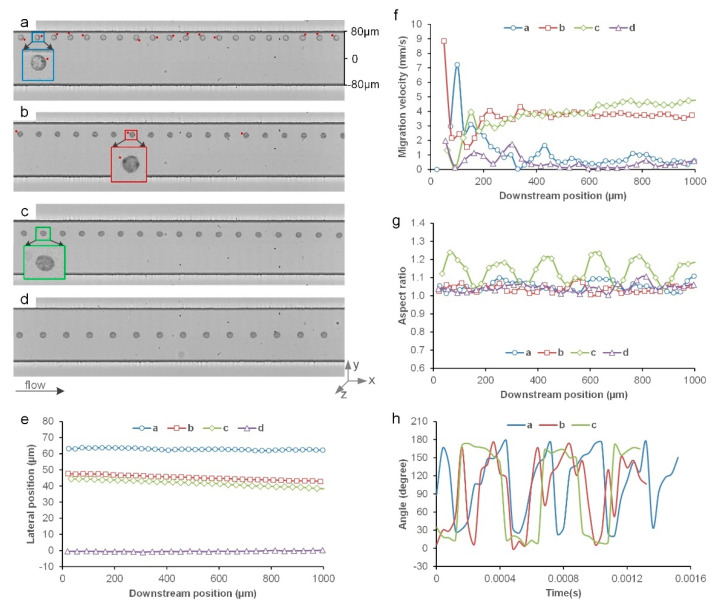
Inertial migration of single cells: (**a**) A single cell near channel sidewall shows very small migration. The red dot indicates the relative position of the dark spot on the cell surface (blowup view in the inset) and shows the rotation of the cell; (**b**) A cell away from the wall migrates toward the channel center. The red dot indicates the rotation of the cell; (**c**) The relatively fast migration of an oval cell; (**d**) A cell close to the channel centerline shows minimal lateral migration; (**e**–**h**) The lateral displacement, lateral migration velocity, aspect ratio, and the orientation of the single cells shown in (**a**–**d**). The bright-field images in (**a**–**d**) are stacked every two frames to show the trajectories of the cells. The angle of the cell in (**d**) was not measured due to the lack of an indicative marker.

The inertial migration of single cells depends on their initial lateral positions, suggesting the spatial heterogeneity of lateral migration (Figure 1). In this straight channel with a rectangular cross-section, there are two final focusing positions located in the centerline near the top and bottom walls. All cells are expected to migrate to these positions eventually [20]. Cells near the sidewalls and close to the channel centerline show very small lateral migration in the 1 mm imaging window. On the contrary, cells initially located between the channel sidewall and centerline appear to have substantial lateral displacement (Figure 1e). For example, the cell in Figure 1b migrated 5 µm toward the channel center, while the lateral displacement of the cell in Figure 1a was less than 1 µm. Calculation of the lateral migration velocity (Figure 1f) found at least a four times higher migration speed of cells in the middle than those near the sidewalls or centerline. Despite the similarity, we expect the migration velocity to increase for the cells near the channel sidewall and to reach zero for the cells near the channel centerline, considering the latter is approaching the final equilibrium position based on our two-stage model [20]. As the model suggests a significant role of cell rotation in migration, we also examined the rotational behavior of the cells when it was possible.

We found that cells rotate in the flow while traveling downstream. As shown in Figure 1a–c, the dark spots on the cell surface clearly change their position in each frame, indicating cells spinning in the flow. For the cell near the sidewall (Figure 1a), the rotation appears mainly about the *z*-axis, as the dark spot is visible most of the time in the top view image. The rotation had components about both the *y*-axis and *z*-axis for the cells in the middle, as suggested by the periodical disappearance of the dark spot on the cell surface in Figure 1b. The rotation about the two axes is noticeable for the elongated cell in Figure 1c and is reflected in the periodical fluctuation of the cell aspect ratio (AR) curve in Figure 1g. Ideally, the AR curve should not change if the rotation is only about one axis for this ellipsoid cell. Since the cells are not perfectly spherical, as indicated by the AR curves, the rotation about the *z*-axis can be measured (Figure 1h). The angular velocity around the *z*-axis was found to be 10,471 rad/s and 12,083 rad/s for the cells in Figure 1a and Figure 1c, respectively. It is worth noting that the ellipsoid cell (19 µm in the major axis, 15 µm in the minor axis, area 242 µm^2^) in Figure 1c had a higher migration velocity than the round-shaped cell (17 µm in diameter, area 226 µm^2^) in Figure 1b despite their similar size (4.7 mm/s vs. 3.7 mm/s, as shown in Figure 1f).

### 3.2. Inertial Migration of Cell Doublets

**Figure 2:**
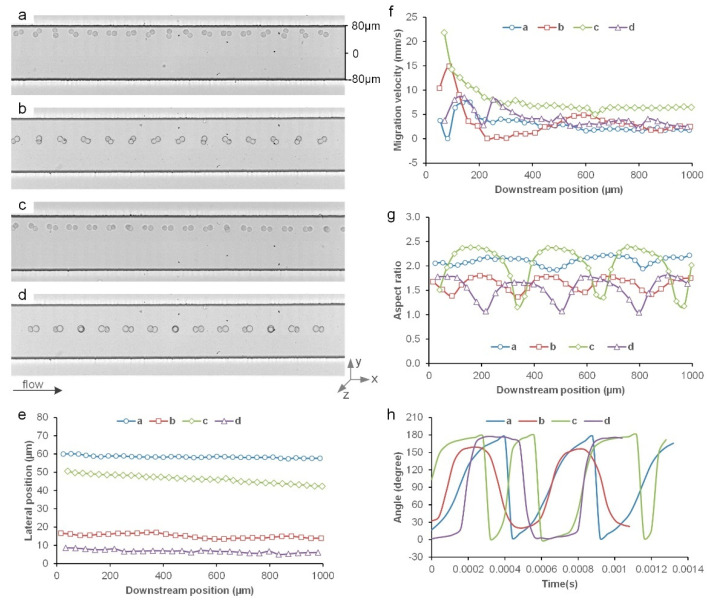
Inertial migration of doublets: (**a**) A cell doublet near channel sidewall slowly migrates away from the wall while rotating about the *z*-axis; (**b**) A cell doublet shows a kayaking motion as it moves downstream the channel; (**c**) A cell doublet is transitioning to tumbling motion (rotating about *y*-axis); (**d**) A cell close to the channel centerline is tumbling downstream; (**e**–**h**) The lateral displacement, lateral migration velocity, aspect ratio, and the orientation of the four doublets in (**a**–**d**). The bright-field images in (**a**–**d**) are stacked every two frames to show the trajectories of the cells.

Like single-cell migration, the lateral migration of cell doublets first accelerates as they move away from the sidewalls, and slows down as they approach the channel center (Figure 2). For example, the lateral displacement of the cell doublet near the sidewall (Figure 2a) was 2.3 µm while the one in Figure 2c migrated more than 8.3 µm (Figure 2e); the calculated migration velocity was 1.8 mm/s and 6.5 mm/s, respectively. Despite that the lateral displacement (2.6 µm) of the cell doublet in Figure 2d was close to that in Figure 2a, it migrated slightly faster due to the higher downstream velocity in the channel center. Overall, these cell doublets migrated at higher lateral velocities as compared to single cells (Figure 1f and Figure 2f). Nonetheless, unlike the largely smooth migration curves in Figure 1e, the curve of the cell doublet in Figure 2b manifests a fluctuation as it travels downstream, which appears to be related to its kayaking rotation pattern [34].

Cell doublets migrate laterally with distinct rotation patterns in the inertial flow (Figure 2). The rotation pattern observed can be generally classified as a tumbling motion or kayaking motion (Figure 2b) [34]. The former includes three subgroups: z-tumbling, which is the rotation around the *z*-axis (Figure 2a); y-tumbling, which is the rotation around the *y*-axis (Figure 2d); and hybrid tumbling, which is the rotation around both the *y*- and *z*-axes (Figure 2c). For the cell doublet near the sidewall, it rotated predominantly around the *z*-axis (z-tumbling) while slowly moving away from the wall (Figure 2a), which is similar to the rigid ellipsoid particles reported previously [35]. On the contrary, the rotation of the cell doublet near the channel center (Figure 2d) was mostly y-tumbling. The different rotation patterns are also evidenced in the measurements of the time-resolved AR (Figure 2g) and the angle change in the major axis of the doublets (Figure 2h). Specifically, z-tumbling shows very small changes in the AR curve (AR stays around two) while pure y-tumbling results in large periodic AR changes (for example, AR cycles between one and two). The hybrid tumbling shall have AR larger than one but smaller than two, ideally. In reality, since the sizes of the two cells in a doublet can be different and the doublets can be stretched in response to the flow shear due to cell deformability, the AR measured can be larger than two, as shown in the green curve in Figure 2g.

The distinct rotation patterns of the cell doublets are reflected in the curves of the measured angle between the major axis of the doublets and the downstream direction (Figure 2h). Due to the way that the angle was measured in ImageJ^®^, the angles are between 0–180°, reflecting half of the full rotation. The rotation about the *z*-axis (z-tumbling) is reflected in the slope of each half rotation while the plateau is representative of the component of y-tumbling, as shown in the blue and purple curves in Figure 2h. While these two curves show repetitive peak profiles, the green curve includes a wide peak and a narrow peak in a full rotation cycle. According to the trajectory in Figure 2c, the former is corresponding to the main rotation about the *z*-axis while the latter is due to the change in the main rotation about the *y*-axis, as suggested by the overlapped cell appearance in the doublet and thus by the dips in the green AR curve (Figure 2g). Hence, this cell doublet switched the rotation axis from the *z*-axis to the *y*-axis alternately (hybrid tumbling). The change in the rotation axis did not affect the overall migration velocity, as indicated by the constantly decreasing lateral position (Figure 2e) and by the largely flat curve of the migration velocity after the initial drop (Figure 2f). However, the kayaking motion reflected in the 20–160° rotation cycles appears to be leading the oscillation in the lateral migration (red curve in Figure 2e).

### 3.3. Inertial Migration of String Triplets

While cell triplets can have different spatial formats, they also migrate toward the channel center, generally following a similar migration behavior to singlets and doublets. Since a triplet has three cells in its system, they can arrange in different ways to give two general spatial formats, namely: (1) a string format where there is a middle cell attaching two other cells on each side; and (2) a triangle format where each cell attaches to two adjacent cells forming a triangle shape. Our high-speed imaging indicates that the rotation behavior of the triplets in these formats can differ significantly, which coincides with slightly different lateral migration speeds. Since the cells were alive and the molecular junctions between the cells might be altered in the flow, the spatial format of a triplet may change in the flow. For example, CTCs and clusters may undergo epithelial–mesenchymal transition (EMT), leading to the loss of cell–cell adhesion because of the switch from E-Cadherin and N-Cadherin [36,37]. Due to these reasons, three cells in a perfectly straight line were rarely observed and the string triplets analyzed in this work are mostly in curved lines.

**Figure 3:**
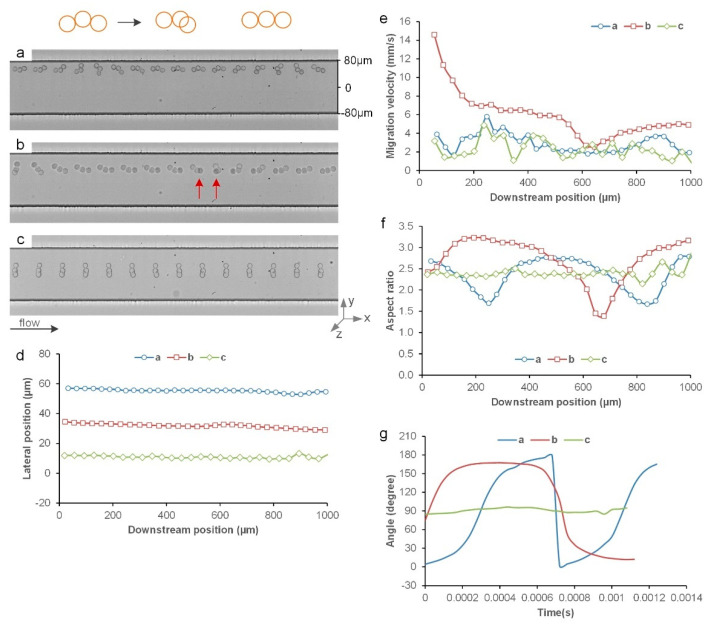
Inertial migration of string triplets: (**a**) A string triplet near channel sidewall slowly migrates away from the wall while rotating about the *z*-axis; (**b**) A string triplet away from the wall slowly migrate toward the channel centerline while folding the cell on its one end. Red arrows indicate the changing of the cell arrangement; (**c**) A string triplet close to the channel centerline is rolling downstream (rotating about *y*-axis) aligning in the direction of channel width (*y*-axis); (**d**–**g**) The lateral displacement, lateral migration velocity, aspect ratio, and rotation of the three doublets in (**a**–**c**). Note that the rotation of the triplet about the *y*-axis was not captured in the green curve in (**g**). The bright-field images in (**a**–**c**) are stacked every two frames to show the trajectories of the cells.

String triplets can have distinct rotation patterns compared to cell doublets (Figure 3a–c). The string triplet near the channel wall (Figure 3a) was rotating about the *z*-axis while traveling downstream, which is similar to the cell doublet in Figure 2a in terms of lateral displacement (Figure 3d) and migration velocity (Figure 3e). However, the spatial arrangement of the three cells in the triplet changed significantly during migration, as indicated in the two large dips in the blue AR curve in Figure 3f. According to Figure 3g, this triplet was rotating much slower than the doublet (4363 vs. 6545 rad/s), despite this string triplet being larger than the doublet in Figure 2a. Similar observations were made for the string triplet in Figure 3b, where a more drastic change in the spatial arrangement was observed at the downstream position of 676 µm. One of the cells in the triplet flipped its horizontal position relative to the other two cells. This change accompanied the reduction in migration velocity and the sharp decrease in the measured AR (Figure 3f). As the string triplet moved closer to the channel center, a new rotation pattern was observed (Figure 3c). This string triplet is uniquely oriented orthogonally to the flow direction and rotated mainly about the *y*-axis. Our recent work [34] suggests this is due to the large blockage ratio (>0.72) and it was identified as a log-rolling motion. Interestingly, this log-rolling triplet had a small migration velocity of 1.5 mm/s.

**Figure 4:**
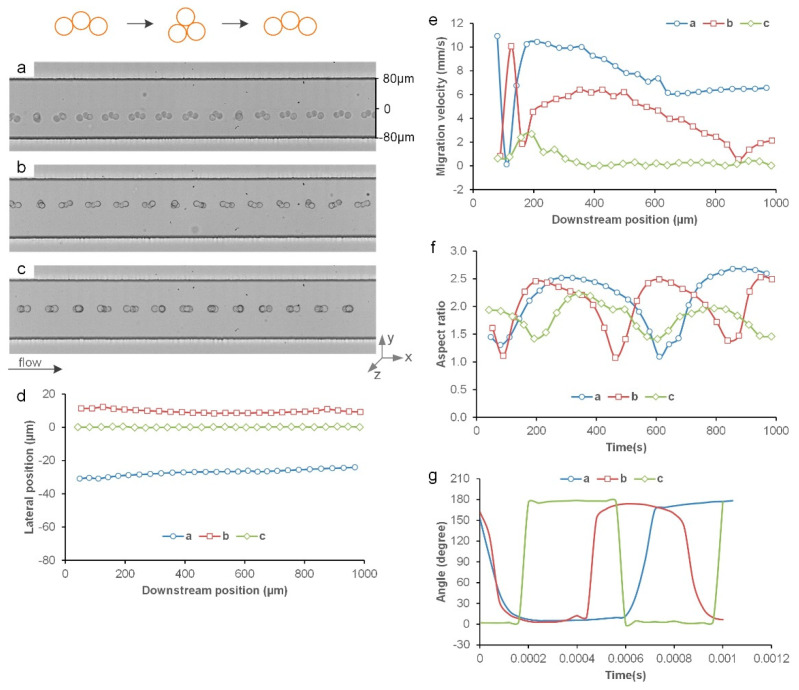
String triplets changing format during migration: (**a**) A string triplet fast migrates toward the centerline while rotating about *y*-axis; (**b**) A string triplet changes its cell arrangement while moves downstream; (**c**) A string triplet close to the channel centerline is tumbling downstream (rotating about *y*-axis) and changing its shape; (**d**–**g**) The lateral displacement, lateral migration velocity, aspect ratio, and rotation of the three doublets in (**a**–**c**). The bright-field images in (**a**–**c**) are stacked every two frames to show the trajectories of the cells.

The string triplet can also change into triangle format momentarily in the flow (Figure 4a–c). The string triplet in Figure 4a changed into the triangle format two times during its fast migration toward the channel center, as shown in the blue curves in Figure 4d,e. In the other two cases, the string triplets changed three times into triangle format, as indicated in the sharp dips in the AR curves (Figure 4f). The migration velocities of these two triplets were smaller than the first triplet as they were closer to the channel center. In all these cases, the rotation about the *z*-axis (z-tumbling) was quite slower than the string triplets in Figure 3. Specifically, the blue curve did not complete a full cycle of rotation about the *z*-axis while the red curve appears to have reached a full cycle. The flat parts of the curves and the occurrence of the triangle triplets suggest that the slow rotation about the *z*-axis led to the transformation of the triplet format. The green curve in this case measures the rotation about the *y*-axis (y-tumbling), as seen in Figure 4c with the treadmill movement. The small fluctuations in the blue and red curves in Figure 4d suggest the transformation impacted the lateral migration; however, how exactly the transformation is affecting the migration cannot be conclusively explained, as the change in the migration velocity did not always occur simultaneously with the change in the aspect ratio (Figure 4e,f).

### 3.4. Inertial Migration of Triangle Triplets

**Figure 5:**
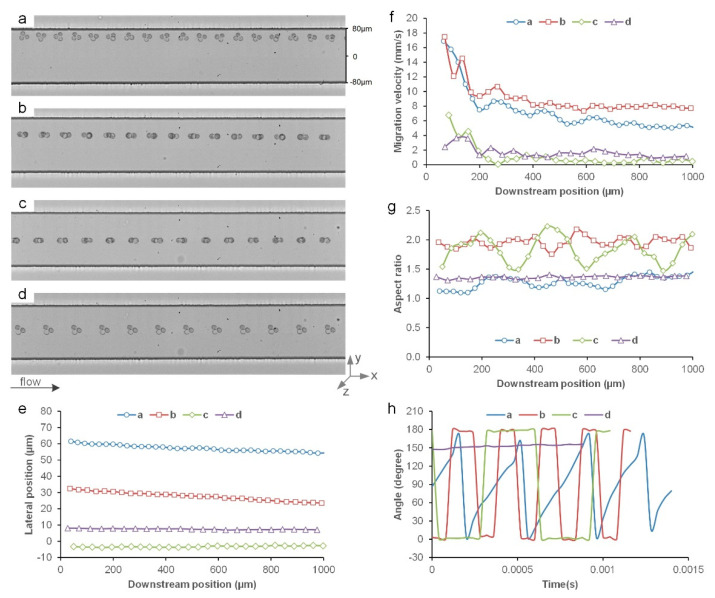
Inertial migration of triangle triplets: (**a**) A triangle triplet near channel sidewall migrates away from the wall while rotating about the *z*-axis; (**b**) A triangle triplet migrates fast toward the channel centerline while tumbling (rotating about *y*-axis); (**c**) A triangle triplet near channel centerline tumbling downstream (rotating about *y*-axis) with very small migration; (**d**) A triangle triplet with little rotation shows very small lateral migration; (**e**–**h**) The lateral displacement, lateral migration velocity, aspect ratio, and the rotation of the four doublets in (**a**–**d**). The bright-field images in (**a**–**d**) are stacked every two frames to show the trajectories of the cells.

Similar to string triplets, the triangle triplets show location-dependent migration and rotation behavior (Figure 5a–d). The triplet near the sidewall (Figure 5a) rotated primarily about the *z*-axis, just like the string triplet (Figure 3a), doublet (Figure 2a), and singlet (Figure 1a). However, this triangle triplet migrated much faster, as indicated by the 7.2 µm lateral displacement in Figure 5e and the 5.3 mm/s average migration velocity at the end (Figure 5f). When the triplet was away from the wall, it changed its primary rotational axis from the *z*-axis to the *y*-axis (Figure 5b) and its lateral migration velocity was slightly higher (7.7 mm/s). The change in the primary rotational axis is also reflected in the periodic fluctuation in its AR curve (Figure 5g) and in the square-wave form-like curve of the angle measurements (Figure 5h) as opposed to the triangle-wave form-like curve for the triplet in Figure 5a. For the triangle triplet near the channel center, it rotated about the *y*-axis (y-tumbling) and very little lateral displacement (0.4 µm) was observed (Figure 5e). This is expected as the triplets are approaching their equilibrium position [20]. However, its rotation about the *y*-axis appears slower than the triplet in Figure 5b.

The rotation appears to be associated with the migration velocity. In a rare case, we found a triangle triplet close to the channel center line (Figure 5d) and its rotation was very small (8.3° during the observed migration, and the angular velocity around the *y*-axis was only 164 rad/s). While its rotation about the *y*-axis could not be measured (the triplet must be flipped horizontally at least once to measure the rotation about the *y*-axis), this rotation was also minimal, if any, according to the high-speed imaging in Figure 5d. This minimal rotation coincided with the observed small lateral displacement of 1 µm (Figure 5e) and thus the small lateral migration velocity of 1.2 mm/s (Figure 5f). On the contrary, the fast-migrating triangle triplet in Figure 5a had a *z*-axis angular velocity of 8267 rad/s with a migration velocity of 5.3 mm/s. Moreover, the doublet located in a similar lateral position in Figure 2d had a higher migration velocity (2.5 mm/s) despite its smaller overall size than the triplet in Figure 5d. We noticed that the angular velocity of the doublet about the *z*-axis was also small but discernible in Figure 2d (~1900 rad/s) and its angular velocity about the *y*-axis was 9817 rad/s. As a result, the migration velocity is related to the rotation in both directions. However, correlation cannot be easily established as the cells in their equilibrium positions have large angular velocities about the *y*-axis due to a large shear rate (Figure 4c and Figure 5c). More work is required to elucidate the relationship between migration and rotation.

### 3.5. Comparison of Single Cells, Doublets, and Clusters

**Figure 6:**
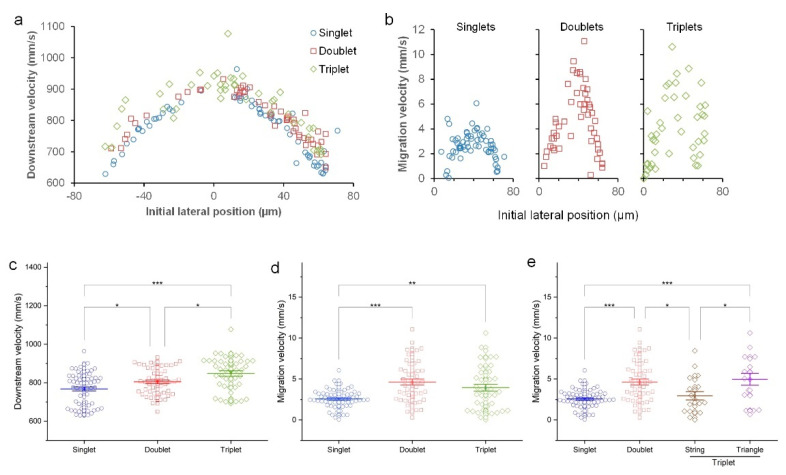
Downstream velocity and lateral migration velocity: (**a**) Downstream velocity of all singlets, doublets, and triplets resembling the parabolic velocity profile of the channel flow; (**b**) Migration velocity of singlets, doublets, and triplets depending on initial lateral position; (**c**) Downstream velocity comparison of single cells, doublets, and triplets; (**d**) Doublets and triplets migrate faster than singlets in general while no significant difference in migration velocity of doublets and triplets are found; (**e**) Triangle triplets migrate faster than string triplets and singlets in general. N = 66, 51, 43, 20, 17 for single cells, doublets, triplets, string triplets, and triangle triplets, respectively. Additionally, 6 of the 43 triplets were excluded from comparison in (**e**) as they were in a hybrid format (changing from string to triangle from time to time). * *p* < 0.05, ** *p* < 0.01, *** *p* < 0.001.

Aggregated data of 160 single cells, doublets, and triplets confirm the location-dependent downstream velocity (Figure 6a) and lateral migration velocity (Figure 6b) in inertial flow. The distribution of the measured downstream velocities of the three groups resembles the parabolic velocity profile of the flow inside a microchannel (Figure 6a), which is reasonable since the fluid drag is responsible for the downstream movement of the cells and clusters [20]. Thus, the downstream velocity increases as the cells approach the channel center. Interestingly, our plot also indicates that the downstream velocity is size-dependent in general (Figure 6a). Overall, the triplets have higher streamwise velocity than doublets and the single cells exhibit the lowest downstream velocity. One-way ANOVA analysis indicates significant differences in the downstream velocities among the three groups (Figure 6c), with the triplets moving much faster than the single cells (10% faster on average). This suggests that triplets were further away from the wall vertically considering the parabolic velocity profile in the channel flow. The broad distribution of the downstream velocity is due to the difference in the lateral and vertical position of cells and clusters. It is worth noting that the triplet with the highest downstream velocity was the one with little observed rotation in Figure 5d.

The lateral migration velocity appears to be dependent on the initial lateral position and the group of cells and clusters. Regardless of the group of the cells and clusters, their migration velocity first increases as they move away from the sidewalls and reaches the maximum roughly in the middle of the channel center and sidewall (Figure 6b) or about 25% of the channel width away from the wall. Thereafter, the lateral migration velocity decreases and eventually drops to zero as the cells and clusters equilibrate in the channel center. As expected, the single cells migrate much slower than doublets and triplets since the inertial migration is size-dependent [20]. The lateral migration velocity of the single cells is mostly below 5 mm/s while many of the doublets and triplets have a velocity above 6 mm/s. Such difference in the lateral migration velocity is confirmed in the one-way ANOVA analysis in Figure 6d.

However, we found that the lateral migration velocity of triplets is unexpectedly similar to the doublets (Figure 6d), which is attributed to the impact of the triplet shape (Figure 6e). No significant difference in migration velocity was found in the doublets and triplets. Since triplets can assume either of the two basic shapes, string or triangle, we further classified the triplets into two subgroups. We found that the migration velocity of the triangle triplets is significantly different from that of the string triplets (*p* < 0.05). Moreover, the migration velocity of triangle triplets is significantly different from the doublets, while no significant difference was found between the string triplets and single cells (Figure 6e). Such findings are rather unexpected, considering that even string triplets are larger than single cells and that the inertial migration is strongly size-dependent. Many factors could contribute to such observations, such as the shape effect, the deformability of the live cells, and the rotation pattern of the cell aggregates. More work is required to elucidate the reasons behind such observations. Additionally, we found that the ratio of the downstream velocity and lateral migration are mostly larger than 100, with minimum ratios of 128, 73, and 78 for the singlet, doublet, and triplet, respectively.

## 4. Concluding Remarks

In this work, we profiled inertial migration at the single cell and single cluster levels, revealing a number of new findings that are directly related to the application of inertial microfluidics. First, inertial migration is spatially heterogeneous and depends on the initial location of the cells or clusters, which is in agreement with our recent results mapping the inertial migration in channel cross-section [33]. Regardless of cell shape or cluster format, the inertial migration velocity first increases before decreasing as cells and clusters move away from the sidewall toward the center. The lateral migration velocity reaches its peak at a lateral position of ~25% channel width regardless of the shape and size of the cell clusters, despite doublets and triplets accelerating faster than single cells. Interestingly, this trend of migration velocity partly resembles the particle migration in whole blood flow that we measured recently [38]. In addition, we found that the larger clusters have higher downstream velocity, which also depends on the lateral position.

Size dependence alone cannot predict the inertial migration of cell clusters and the shape can significantly impact migration. Inertial microfluidics are best known for their strongly size-dependent migration [20,27]. While faster migration of the cell doublets was observed as compared to single cells, no significant difference was found between the migration velocities of doublets and triplets. Moreover, the migration velocity of the triplets in the string format was found to be comparable to the single cells, even though their sizes are quite different. Meanwhile, the triangle triplets migrated faster than the single cells but not the doublets. Early work suggests that the rotation of rod-like particles can lead to slightly different and shape-dependent focusing positions [35]. Here we show that the shape or the format of the cluster can negatively impact the migration velocity.

The rotation of the cells and clusters is involved inlateral migration but its contribution is complicated and remains elusive. The cells and clusters near the sidewalls rotate primarily about the *z*-axis and those near the channel centerline rotate mostly about the *y*-axis. Both the *y*-axis and *z*-axis rotational components are present for those in between the sidewall and the channel centerline. Our results suggest that rotation is required for lateral migration and at the same time fast rotation does not lead to fast migration. The triangle triplet showed very little migration while the rotation was minimal. On the other hand, fast rotation about the *y*-axis in the equilibrium position yields no migration. More work is required to fully understand the role of rotation in inertial migration. Computational modeling can be handy in this case, as the rotation can be artificially modified in simulations [34].

Apart from the improved understanding of migration, these findings will have profound implications for the applications of inertial microfluidics. Owing to the strong size-dependent migration, inertial microfluidics have been widely used in label-free separation of particles and cells, such as blood sorting and isolation of CTCs. We [21,23] and others [26,27,28,29] have demonstrated the separation of the larger CTCs and CTC clusters from patient blood using inertial microfluidics. In light of the discovery of the CTC clusters, which are of great clinical significance [6,9,39], the separation of single cells and clusters will be of great use in improving the understanding of cancer metastasis and cancer cell biology. Based on our findings, the separation of single cells from cell doublets is readily achievable while separation of the triplets from single cells can be very challenging. On the other hand, our results also suggest that sorting the clusters based on the shape is possible in inertial microfluidic devices, for example, the separation of string triplets from triangle triplets, which can be useful in the investigation of inter-cellular communications. Since the shape can be negatively correlated to the migration velocity, this shape effect needs to be considered where the separation of non-spherical particles is concerned.

With our new findings, we also notice a few caveats. First, we used live cells and clusters in this work aiming to provide insight into the migration behavior of living real-world CTC clusters, to help the applications of processing biological samples that cannot be fixed. These live cells and clusters may respond to the flow stress differently from fixed cells. The string triplets could change into triangle triplets as they were soft and flexible. Therefore, the results reported in this work include the effect of the compliance of living cells, which may also reflect the impact of intracellular molecular events such as EMT. Additionally, we tested the inertial migration in one device using one flow rate. Thus, the results might not reflect the full picture of cluster migration. This is solely due to the significant effort and resources required to manually analyze the data at a single cell level. In our future work, we will use machine learning and computer vision to automate the analysis, which will allow us to investigate more conditions including the flow rate, channel geometry, and cell types. Nonetheless, our findings significantly contribute to the understanding and biomedical applications of inertial migration involving complex bioparticles.

## Data Availability

Data available upon request.
